# A Case of Jejuno-Jejunal Intussusception Caused by Underlying Metastatic Melanoma

**DOI:** 10.7759/cureus.36217

**Published:** 2023-03-16

**Authors:** Hardeep S Ahdi, David Kruchko, Nahren Asado, Samir Kakodkar

**Affiliations:** 1 Internal Medicine, Advocate Lutheran General Hospital, Park Ridge, USA; 2 Gastroenterology and Hepatology, Advocate Lutheran General Hospital, Park Ridge, USA; 3 Pathology, Advocate Lutheran General Hospital, Park Ridge, USA; 4 Department of Medicine, Division of Gastroenterology, Advocate Lutheran General Hospital, Park Ridge, USA

**Keywords:** small bowel melanoma, jejunal intussusception, intestine intussusception, malignant melanoma metastasis, jejunojejunal intussusception

## Abstract

Intussusception in adults is a rare finding with a majority of cases occurring in the pediatric population. It occurs infrequently and its presentation, etiology, and treatment differ from childhood intussusception. When discovered in adults, it raises suspicion for a neoplastic process serving as the pathological lead point. Cross-sectional imaging is the primary study of choice for diagnosis, but at times, a more invasive approach involving an exploratory laparotomy is required posing an increased risk for morbidity and mortality. Here we present a 64-year-old male who was found to have jejunal-jejunal intussusception that was surgically removed with pathology revealing metastatic melanoma as the lead point. This case highlights a unique presentation of a melanoma that was previously eradicated with immunotherapy and now had metastasized to the intestine many years later.

## Introduction

Intussusception is a condition in which a proximal portion of the intestine invaginates into its distal aspect, a phenomenon known as telescoping [1]. It is commonly found among pediatric patients and infants with a peak incidence of 5-7 months of age in infants and 74 per 100,000 in children under one year of age [1]. Although the diagnosis can be delayed because of its longstanding, intermittent, and non-specific symptoms, typical signs include colicky abdominal pain, mucus or blood-tinged stool, diarrhea, and emesis [1, 2]. Intussusceptions in adults are usually due to benign neoplastic lesions, with gastrointestinal metastasis of cutaneous malignant melanoma as the culprit in no more than 15% of cases [3]. The most sensitive diagnostic method in the early detection of intussusception is an abdominal computed tomography (CT). Adult intussusception necessitates surgical intervention due to a high incidence of malignancy. The prognosis is generally poor, given this disease’s rarity and malignancy prevalence. Only through early recognition of diagnosis and timely involvement of a multidisciplinary team can it help reduce complications associated with high morbidity and mortality. Here we present an interesting case of a patient who was found to have intussusception caused by a relapsed metastatic melanoma.

This article was previously presented as a meeting abstract at the American College of Gastroenterology Annual Scientific Meeting from October 21-26, 2022.

## Case presentation

**Figure 1:**
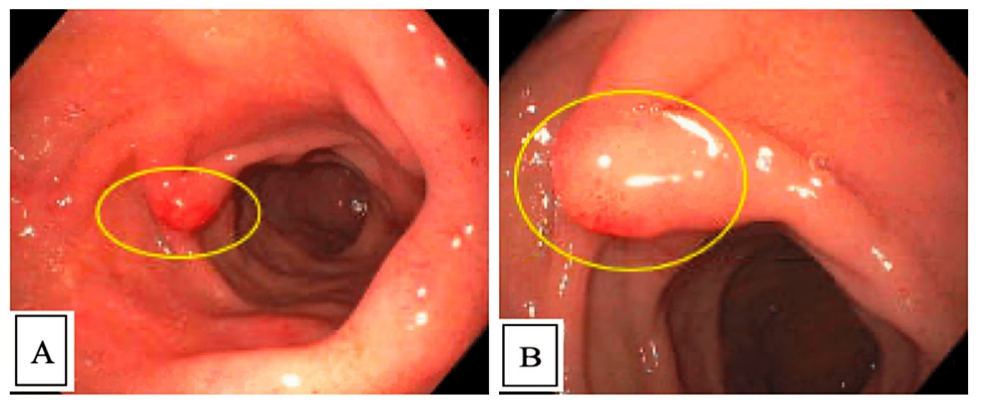
Esophagogastroduodenoscopy (EGD) findings A: Duodenal bulb, duodenitis and prominent bulb with erythema (circle) B: Duodenal bulb, closer view of prominent duodenal fold in bulb (circle)

**Figure 2:**
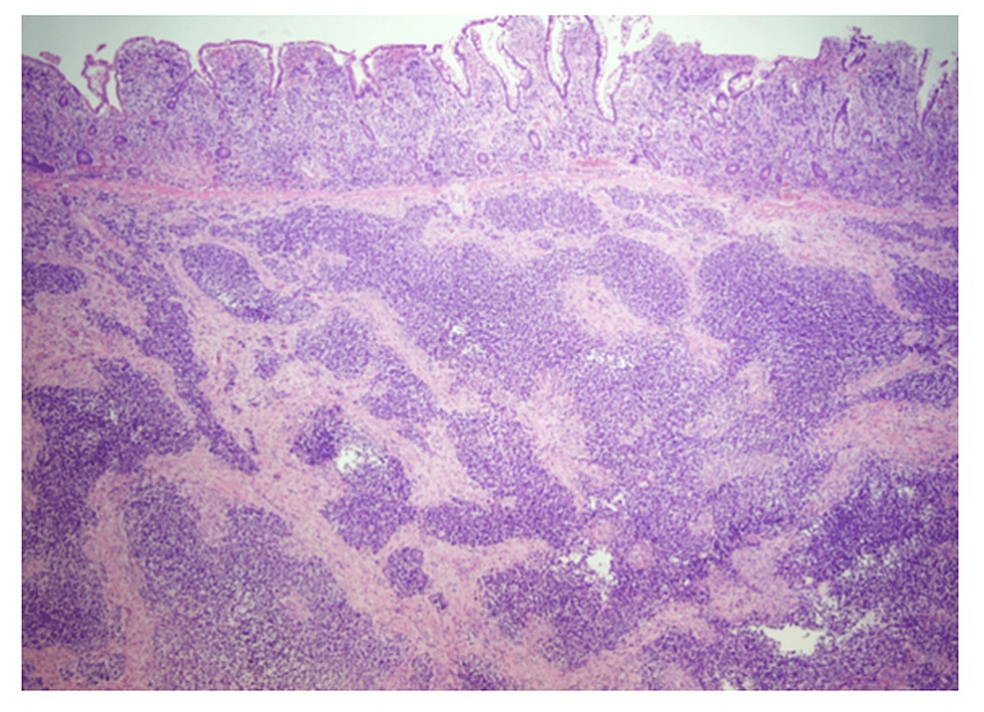
Histopathology exam image This image illustrates nests of tumor cells diffusely invading into the mucosa (top), submucosa (mid), and muscularis propria (bottom) layers (40X; hematoxylin and eosin).

**Figure 3:**
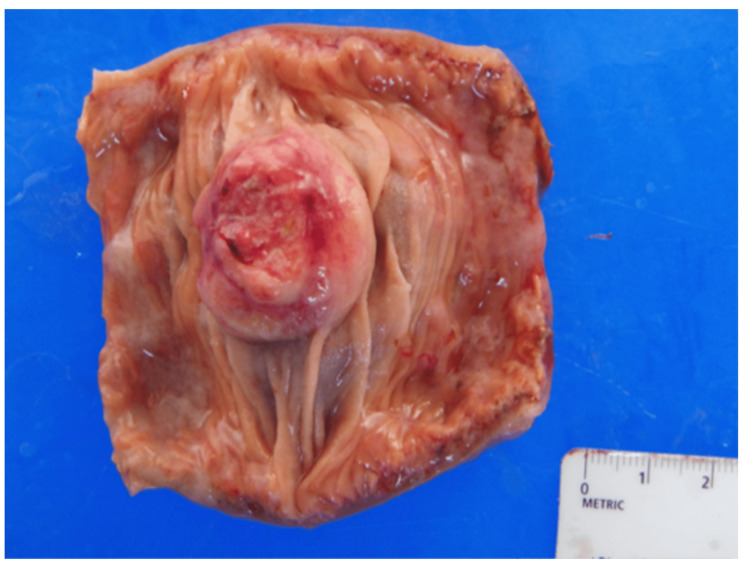
Surgical specimen image This image illustrates the opened and resected small intestine specimen, measuring a 3.1 cm x 2.7 cm umbilicated mass extending into lumen.

**Figure 4:**
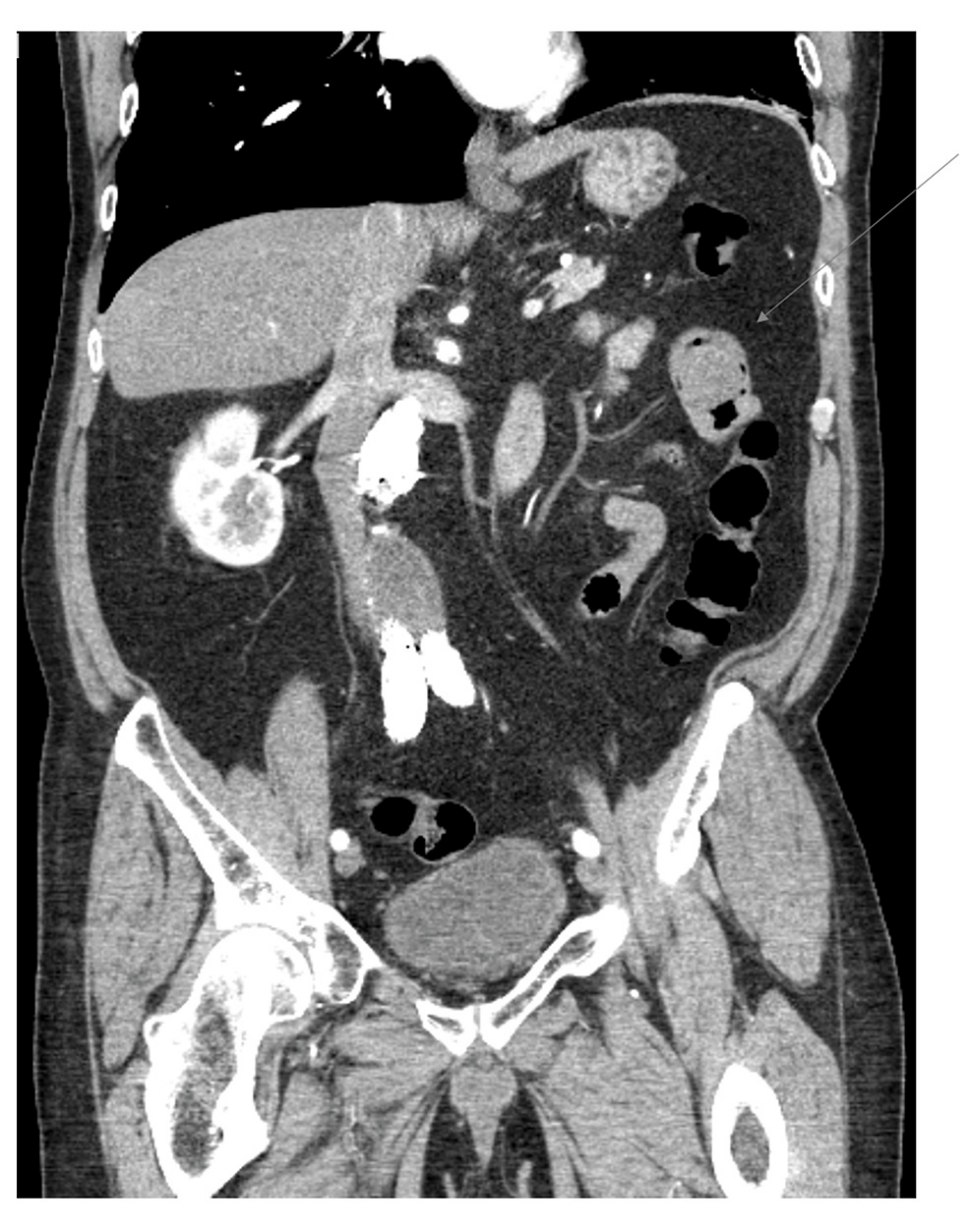
Computed tomography angiography of abdomen and pelvis, coronal plane This image illustrates an arrow pointing to the metastatic lesion with surrounding air viewed in the coronal plane.

**Figure 5:**
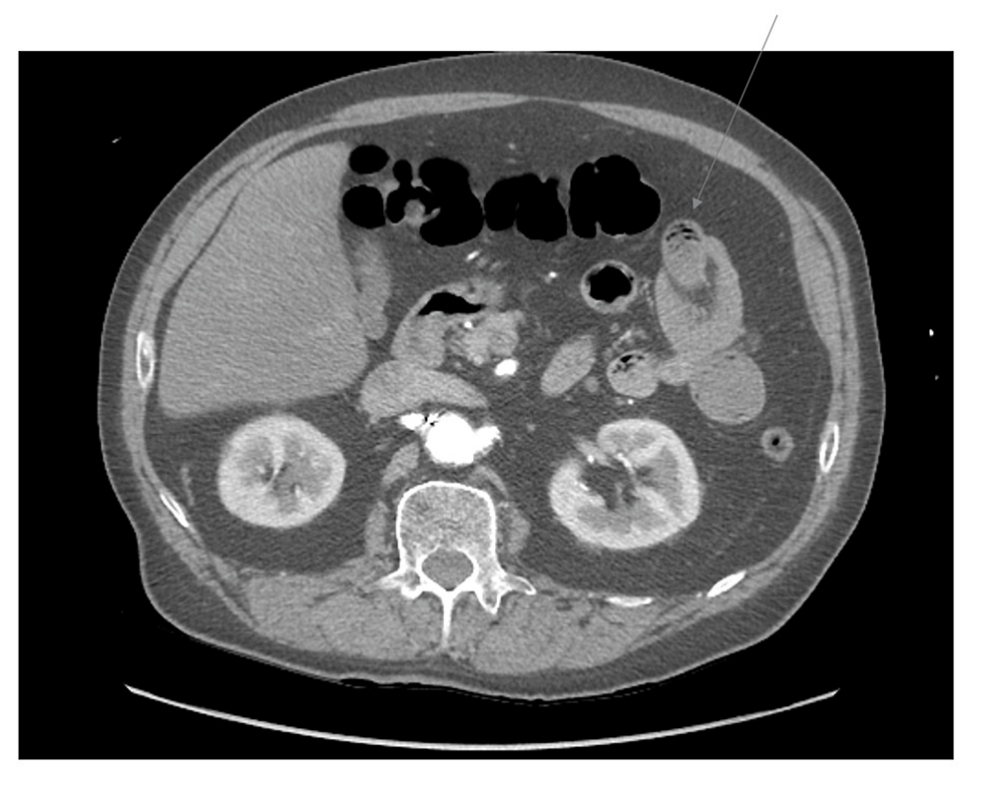
Computed tomography angiography of abdomen and pelvis, axial plane This image illustrates an arrow targeting the intussusception viewed in axial plane.

A 64-year-old male with a history of alcoholism, 94 pack-years of smoking, coronary artery disease on aspirin 81 mg daily, abdominal aortic aneurysm repair, and stage IV melanoma presented with four days of melena and abdominal discomfort. His melanoma was initially diagnosed in 2010 via punch biopsy of a left thigh lesion and was treated with wide local excision. His follow-up lymph node scan was negative for metastasis. Approximately five years later he was diagnosed with widely metastatic malignant melanoma. He was hospitalized during that time for diffuse pain and ascites with skeletal and visceral mets, including the peritoneum. He received radiation therapy to the skull and was started on vemurafenib with near complete response. Serial PET imaging suggested progressive disease and he was transitioned to Keytruda. He was started on Xgeva in light of the bone mets. An MRI brain was negative in 2019. He had been off therapy for five years, with negative PET scans two years prior. On arrival, his vital signs showed that he was afebrile, tachycardic 112, tachypneic 23, BP 130/88, and saturating well on room air. Physical exam revealed conjunctival pallor and mild abdominal tenderness to palpation without rebound or guarding. Labs revealed hemoglobin (Hgb) 10.7 g/dL (seven months prior noted to be 15.1 g/dL), platelet count 253,000/µL, aspartate aminotransferase (AST) 40 U/L, alanine transaminase (ALT) 63 U/L, blood urea nitrogen/creatinine (BUN/Cr) 26 mg/dL, and international normalized ratio (INR) 1.0. His last colonoscopy 14 years prior demonstrated multiple tubular adenomas. He received a dose of IV Protonix 40 mg and was started on a Protonix drip given concerns for upper GI bleeding. An upper endoscopy showed mild duodenitis, as shown in Figure 1. Gastric biopsies were negative for H. pylori, and duodenal biopsies suggested gastric metaplasia and Brunner’s glands hyperplasia. Given his vascular risk factors, computed tomography angiography (CTA) was obtained which revealed a jejuno-jejunal intussusception with focal wall thickening measuring approximately 5.3 cm in length, for which an underlying lesion was difficult to exclude, as shown in Figures 2, 3. There were no signs of bowel obstruction. He underwent exploratory laparotomy with findings of an 8 cm intussuscepted bowel with a lead point mass that was resected with pathology revealing metastatic melanoma 3.1 cm x 2.7 cm involving the entire wall of the small bowel, as shown in Figures 4, 5. Surgical report revealed that the small bowel was identified to run distally to the ileocecal valve proximally towards the ligament of Treitz where the intussusception was encountered in the proximal jejunum. The intussusception reduced with elevation of the bowel. Following the ex-laparotomy, the patient was treated supportively with pain control and supplemental oxygen and periodic incentive spirometer to prevent postoperative atelectasis and to help assist with lung ventilation. His postoperative course was complicated by a mild degree of acute blood loss anemia with an Hgb drop to 7.1 before improving up to 8.6 prior to discharge.

## Discussion

Intussusception can be referred to as the invagination or “telescoping” of a proximal segment of the gastrointestinal tract into the lumen of an adjacent segment. It is predominantly due to the peristaltic contractions constricting and relaxing the lumen at the transition point [1]. Bowel intussusception is commonly seen in children but is rare in adults, accounting for 5% of all cases of intussusception and 1% of all cases of bowel obstruction in adults [4]. The incidence of intussusception varies among age groups. Whereas intussusception in children is often idiopathic where the lead point is thought to be due to lymphoid hyperplasia following a viral infection, about 90% of adult intussusceptions are usually associated with a pathologic lead point from underlying malignancy. Adult intussusception can localize to the small bowel in 52% of the cases [5].

The diagnosis of intussusception can be delayed and oftentimes missed without imaging, and the clinical picture can be challenging due to its variable presentation, often associated with its longstanding non-specific symptoms including abdominal pain, nausea, and vomiting [6]. The classic presentation of intussusception involves repeated bouts of non-specific abdominal pain, vomiting, and currant jelly stools, although this is rarely seen in adults [6]. Although a rare finding, adult intussusception has the potential to cause severe complications if not recognized and treated early, including but not limited to bowel obstruction, necrosis, and perforation [7]. A proposed hypothesis is that as a part of the bowel segment telescopes into an adjacent segment, the mesenteric vasculature supplying the affected bowel can become compressed and thus compromised, resulting in ischemia of the bowel wall [4]. The gold standard for diagnosing intussusception and small bowel obstruction (SBO) is an abdominal computed tomography (CT). It is the most sensitive test because of its capability of identifying a possible lead point causing the intussusception [8]. Our patient presented with a few days of melanic stools and abdominal pain and underwent CTA given his underlying vascular-related risk factors, revealing the jejunal-jejunal intussusception. Following exploratory laparotomy, it was determined to be caused by an underlying metastatic melanoma lesion involving the entire wall of the small bowel in the absence of obstruction-related symptoms.

A systematic review and meta-analysis by Hong et al. showed that a metastatic carcinoma acted as a culprit in the majority of intussusception cases [9]. Known for its high metastatic potential, malignant melanoma has the tendency to metastasize to the gastrointestinal tract as the third most common site, with the small intestine commonly affected which is largely attributed to its rich blood supply [10-12]. Despite this, metastatic melanoma causing small bowel obstruction is relatively rare [3]. Unfortunately, about 50% of all patients treated for melanoma will eventually relapse, with about 50% in regional lymph nodes, 30% with distant metastases, and 20% being local [13]. Although most cases of relapsed melanoma appear to occur within the first few years following treatment, up to 2.4% of cases were noted to recur more than 10 years after treatment [12,13].

The current literature review suggests that there are no clear guidelines on reducing and manipulating an intussuscepted bowel prior to surgical resection. In fact, Aydin et al. performed a case series and were able to demonstrate success with utilizing a conservative approach when exam and imaging findings suggested a self-limited intussusception given a low probability of malignancy acting as a lead point and ischemia [14]. Interestingly, Lvoff et al. also found that an intussuscepted bowel length of <3.5cm was likely self-limiting based on a study of 37 cases of adult intussusception [15]. In comparison to our patient, not only did he have multiple notable risk factors including vascular and cardiac disease, chronic smoking history with active status, and malignancy history, but he also was found to have an intussusception up to 5.3cm in length, deeming an appropriate candidate for emergent laparotomy. Ultimately, surgical resection of malignant melanoma of the GI tract performed laparoscopically or open technique remains the treatment of choice [12, 16].

## Conclusions

Melanoma is one of the most rapidly progressive cancers. Metastasis of cutaneous malignant melanoma causing intussusception is a unique finding with few causes reported. Intussusception in adults can present with non-specific symptoms, and metastatic disease should be on the differential in any patient with a history of melanoma. Early detection increases the likelihood that the cancer is amenable to treatment (i.e., surgical resection) and exploratory laparotomy should be performed promptly in the proper clinical context. In our case, a metastatic melanoma that was treated with immunotherapy approximately greater than 10 years prior was the ultimate cause.
